# Infrequent Pattern Detection for Reliable Network Traffic Analysis Using Robust Evolutionary Computation

**DOI:** 10.3390/s21093005

**Published:** 2021-04-25

**Authors:** A. N. M. Bazlur Rashid, Mohiuddin Ahmed, Al-Sakib Khan Pathan

**Affiliations:** 1 School of Science, Edith Cowan University, Joondalup, WA 6027, Australia; a.rashid@ecu.edu.au (A.N.M.B.R.); mohiuddin.ahmed@ecu.edu.au (M.A.); 2 Department of Computer Science and Engineering, Independent University, Dhaka 1229, Bangladesh

**Keywords:** infrequent, rare, pattern detection, network traffic, unsupervised, feature selection, evolutionary computation, cooperative co-evolution

## Abstract

While anomaly detection is very important in many domains, such as in cybersecurity, there are many rare anomalies or infrequent patterns in cybersecurity datasets. Detection of infrequent patterns is computationally expensive. Cybersecurity datasets consist of many features, mostly irrelevant, resulting in lower classification performance by machine learning algorithms. Hence, a feature selection (FS) approach, i.e., selecting relevant features only, is an essential preprocessing step in cybersecurity data analysis. Despite many FS approaches proposed in the literature, cooperative co-evolution (CC)-based FS approaches can be more suitable for cybersecurity data preprocessing considering the Big Data scenario. Accordingly, in this paper, we have applied our previously proposed CC-based FS with random feature grouping (CCFSRFG) to a benchmark cybersecurity dataset as the preprocessing step. The dataset with original features and the dataset with a reduced number of features were used for infrequent pattern detection. Experimental analysis was performed and evaluated using 10 unsupervised anomaly detection techniques. Therefore, the proposed infrequent pattern detection is termed *Unsupervised Infrequent Pattern Detection (UIPD)*. Then, we compared the experimental results with and without FS in terms of true positive rate (TPR). Experimental analysis indicates that the highest rate of TPR improvement was by *cluster-based local outlier factor (CBLOF)* of the *backdoor* infrequent pattern detection, and it was 385.91% when using FS. Furthermore, the highest overall infrequent pattern detection TPR was improved by 61.47% for all infrequent patterns using *clustering-based multivariate Gaussian outlier score (CMGOS)* with FS.

## 1. Introduction

The current digital ecosystem, bolstered by the innovations and advancements of new technologies produces a massive amount of data continuously. The devices and technological settings that generate the data include the sensor networks, Internet of Things (IoT), healthcare, cybersecurity, and many other domains [1,2,3]. The massive amount of generated data is termed *Big Data*. In the existing literature, we find that several *V*s are associated with the characteristics of Big Data. The most common *V*s are volume, velocity, and variety. These *V*s indicate the amount of data generation, the different types of data, and the speed of data generation [4,5]. Big Data provides the opportunity to the research community to discover new knowledge, such as exploring the identification of different types of network attacks in cybersecurity. However, analysis of data generated by different network applications is computationally expensive [6]. One of the common data analysis tasks in cybersecurity domain is anomaly detection, which basically identifies data patterns, i.e., a pattern-driven data mining process, that identifies data or events that deviate from the usual or expected behavior [7]. To learn, predict, detect, and classify anomalous data in this context, both supervised and unsupervised *machine learning (ML)* anomaly detection techniques are usually used [2]. When the datasets of various domains are considered, including cybersecurity datasets, we observe that they consist of many features. However, not all the features are relevant, i.e., most are irrelevant, as such may degrade the classification or clustering performance by many ML algorithms [2,4]. *Feature selection (FS)* is an approach to select the most relevant subset of features while removing the irrelevant features, which results in reducing the execution time (ET) and improving the classification or clustering performance. Hence, removing unnecessary features in cybersecurity datasets is an essential preprocessing step before the main data analysis task. In addition, FS can also reduce the storage requirements [2,6]. Data fusion usually refers to integrating information gathered from different sources, for example, sensors, IoT, databases, etc. Data fusion also corresponds to collecting different types of data via a procedure and is used for building a model. Data fusion can be categorized into a low, intermediate, or high level that depends on the processing stage. A low-level data fusion can combine different sources of raw data to create new raw data [8,9]. Therefore, data fusion is another necessary step before applying an FS technique or anomaly detection technique to identify a data pattern.

In the literature, many FS approaches have been studied. The *evolutionary computation (EC)*-based FS approaches are widely used as a search technique for the FS process. However, when the search space increases because of the Big Data characteristics, traditional EC-based approaches are not suitable [2,4,6]. Recently, cooperative co-evolution (CC), a meta-heuristic-based FS approach, has shown its effectiveness in Big Data Analytics. Examples of such CC-based approaches include a *cooperative co-evolutionary algorithm-based feature selection (CCEAFS)* [4] with a penalty-based wrapper objective function and a *cooperative co-evolution-based feature selection with random feature grouping (CCFSRFG)* [6]. The terms, *cooperative co-evolutionary algorithm* and *cooperative co-evolution* indicate the same algorithm. CCFSRFG is more effective than CCEAFS. Thus, CCFSRFG can be used as the preprocessing step in cybersecurity data analysis, i.e., for anomaly detection.

This paper introduces a novel infrequent pattern detection by the FS approach, CCFSRFG. The proposed approach has been evaluated by unsupervised anomaly detection techniques. Hence, it is called *Unsupervised Infrequent Pattern Detection (UIPD)*. A benchmark and widely used cybersecurity dataset has been collected from the UNSW Canberra Cyber Centre repository (https://www.unsw.adfa.edu.au/unsw-canberra-cyber/ (accessed on 16 April 2021)). Ten (10) unsupervised anomaly detection techniques have been used to detect infrequent patterns from this dataset. Comparative experimental results analysis indicates that in terms of *true positive rate (TPR)*, in most cases, the proposed infrequent pattern detection approach outperforms the standard state-of-the-art anomaly detection techniques when using FS as a preprocessing step.

### 1.1. Research Questions

This paper aims at answering the following fundamental and associated subquestions:

- How can a feature selection process be applied to the cybersecurity datasets that can select a suitable subset of features and can improve the unsupervised pattern/anomaly detection techniques’ performance?
  – How can the unsupervised pattern/anomaly detection techniques be applied to the original dataset and the dataset with fewer features?
  – Can the infrequent pattern/anomaly detection techniques perform well on a dataset with feature selection as on the original dataset?

### 1.2. Paper Roadmap

The rest of the paper is organized as follows. Section 2 discusses the infrequent pattern mining for network traffic analysis. Section 3 presents the feature engineering approach using evolutionary computation. Section 4 illustrates the proposed methodology for infrequent pattern detection for network traffic analysis. Section 5 contains experimental results and analysis based on a benchmark cybersecurity dataset. The conclusion and future work directions are included in Section 6.

## 2. Infrequent Pattern Mining

A data pattern *X* can be defined as *frequent* if it supports many regular patterns, which correspond to the "common features" in the dataset. On the other hand, a data pattern *X* can be defined as *infrequent* or *rare* whose frequency of appearance is below a user-defined threshold limit in the dataset [10]. Anomaly detection is related to identifying the interesting data patterns, which unusually deviate from their expected behavior. Sometimes, anomaly detection can also be called outlier detection [11]. It is an important data analysis task in many domains, such as cybersecurity, healthcare, the Internet of Things (IoT), fraud detection, and intrusion detection. For example, a cyberattack is a malicious attack that may damage a computing system via unauthorized network access, code, or data injection. Then, anomalies that can be considered can be categorized into three different types: (1) *point/rare anomaly*, (2) *contextual anomaly*, and (3) *collective anomaly*. The first category indicates a specific data instance that deviates from the normal pattern, the second category points to a data instance that behaves anomalously in a specific context, and the third category represents a collection of data instances that behave anomalously [7,12]. The fundamental cyberattacks include: (1) *denial of service (DoS)*, (2) *probe*, (3) *user to root (U2R)*, and (4) *remote to user (R2U)*. DoS interrupts the normal computing and causes unavailability of services, probe attacks a targeted host or network for reconnaissance purpose, U2R tries to get illegal access to an administrative account, and R2U tries to get local access to a targeted system. In the literature, U2R and R2U are grouped into point/rare anomalies, DoS is grouped into collective anomalies, and the probe is grouped into contextual anomalies [5,7].

**Figure 1:**
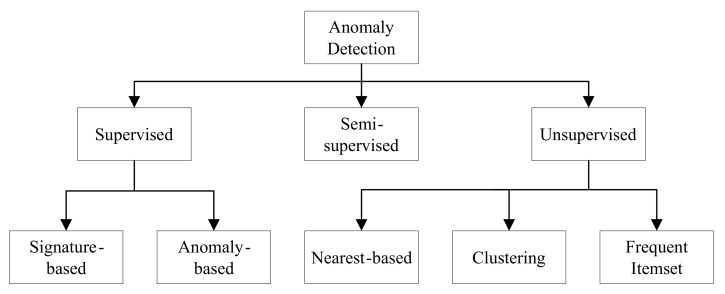
A taxonomy of anomaly detection approaches.

In the case of network traffic analysis, it can be observed that there are several infrequent or rare anomalies in the cybersecurity datasets. We find both frequent and infrequent pattern minings, which have been studied. However, infrequent pattern mining is more challenging than frequent pattern mining [10]. A number of different anomaly detection approaches are used to analyze the network traffic. Three dominant approaches have handled the network traffic analysis tasks: (1) supervised, (2) semi-supervised, and (3) unsupervised [11,12,13]. The anomaly detection techniques, which rely on labeled training data, are supervised. Supervised techniques require training data that are usually expensive to generate. These techniques face difficulties when it comes to detecting new types of attacks. Semi-supervised methods require a small amount of labeled data for building a model to detect anomalies. However, unsupervised techniques do not need any training data and can detect previously unseen attacks [11]. A taxonomy of anomaly detection techniques [11,12] is shown in Figure 1.

**Figure 2:**
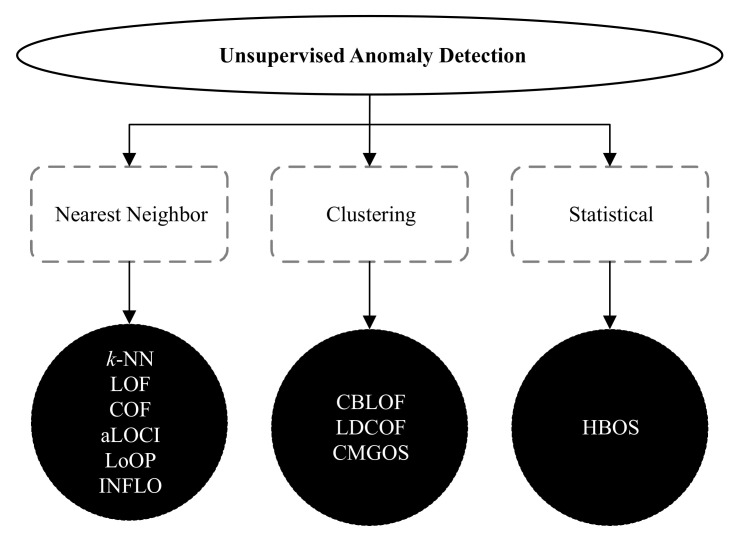
A taxonomy of unsupervised anomaly detection techniques.

There are many unsupervised anomaly detection techniques available in the literature. However, the widely used 10 unsupervised methods fall into three categories: nearest neighbor, clustering, and statistical. These techniques are *k*-NN global anomaly score, local outlier factor (LOF), connectivity-based outlier factor (COF), approximate local correlation integral (aLOCI), local outlier probability (LoOP), influenced outlierness (INFLO), cluster-based local outlier factor (CBLOF), local density cluster-based outlier factor (LDCOF), clustering-based multivariate Gaussian outlier score (CMGOS), and histogram-based outlier score (HBOS) [7]. The taxonomy of all these unsupervised anomaly detection techniques [11,12] is illustrated in Figure 2.

## 3. Feature Engineering Using Evolutionary Computation

**Figure 3:**
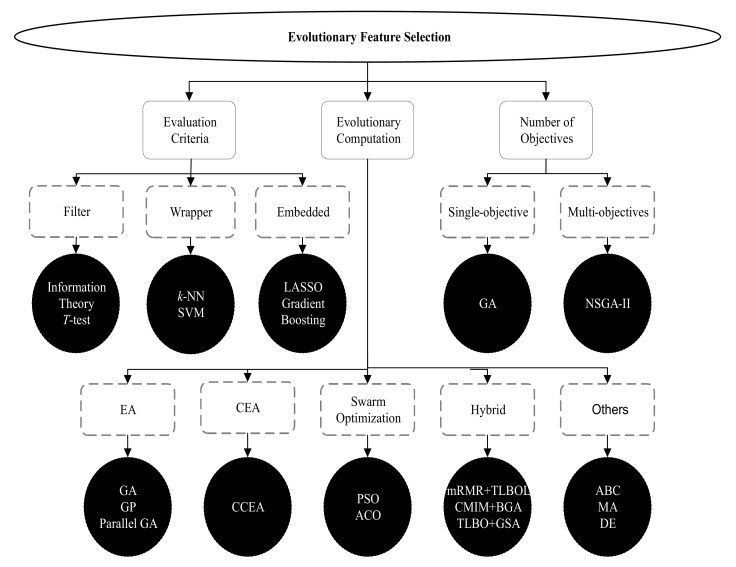
A taxonomy of evolutionary feature selection approaches.

*Feature selection (FS)* or *feature engineering (FE)* is a technique to select a suitable subset of relevant features for representing the datasets with a reduced number of features that can maximize the classification accuracy [2]. Formally speaking, FS is a mechanism for selecting a subset of *s* features from a complete set of *n* features (s<n and *n* is the dimension of the dataset in terms of the number of features) by removing the irrelevant or unnecessary features [14]. Irrelevant features may degrade the classification performance of machine learning (ML) algorithms. Hence, removing these unnecessary or irrelevant features and representing the dataset with fewer features is the primary objective of an FS process. In order to discover the subset of features from the dataset, a search technique is required to initiate the FS process. Different ML algorithms then evaluate the selected subset of features in terms of performance measures, such as classification accuracy. To terminate the FS process, a termination condition, for example, a maximum number of generations or the desired number of features selected, is required to complete the entire process. At the end of the FS process, a validation procedure may test the validity of the selected subset of features in terms of a real-world scenario [2,4]. A range of search techniques, such as heuristics or evolutionary computations (ECs), can be used in the FS process. However, the widely used search strategy in the FS process is EC. A taxonomy of evolutionary FS approaches [2,4,6] is illustrated in Figure 3. Evolutionary FS approaches can be categorized into three types: (1) evaluation criteria-based, (2) evolutionary computation-based, (3) the number of objectives-based. Evaluation criteria-based FS approaches are further categorized into the filter, wrapper, and embedded methods. Filter method scores and ranks data samples using different measures, such as information theory or *T*-test. The wrapper method first selects subsets of features and evaluates the goodness of the selected features using various measures, such as support vector machine (SVM). The embedded method combines both filter and wrapper approaches, i.e., model formation and evaluation of features are performed in a single process. The different EC algorithms that are used in the FS process are evolutionary algorithm (EA), co-evolutionary algorithm (CEA), swarm optimization, hybrid, and other algorithms. The standard algorithms in these categories are genetic algorithm (GA), genetic programming (GP), parallel GA, cooperative co-evolutionary algorithm (CCEA), particle swarm optimization (PSO), ant colony optimization (ACO), minimum redundancy maximum relevance (mRMR), teaching learning-based algorithm (TLBO), TLBO with opposition-based learning (TLBOL), conditional mutual information maximization (CMIM), binary genetic algorithm (BGA), gravitational search algorithm (GSA), artificial bee colony (ABC), memetic algorithm (MA), and differential evolution (DE) [2,4,6].

### 3.1. Cooperative Co-Evolution

The *cooperative co-evolution (CC)* is a metaheuristic algorithm. It is also one kind of evolutionary computation approach and population-based search approach. Potter and De Jong first introduced the CC concept in 1994 to solve large-scale and complex optimization problems [15]. CC follows a divide-and-conquer strategy to divide a large and complex problem into several subproblems. It evolves co-adapted subproblems on an iterative basis to build a complete solution to the problem. Formally speaking, a CC technique decomposes an *n*-dimensional problem of search space S=1,2,…,n into *m* subproblems $S_{1},S_{2},\ldots ,S_{m}$ [15]. Each subproblem with a maximum of *n*-dimensions represents a new search space $SP^{\left(i\right)}$ for a particular problem. In contrast, the rest of the dimensions $n_{j}$, with $j\neq S_{i}$ are kept fixed. Other subproblems follow the same process to decompose the entire search space with lower dimensions, which can be evolved by any population-based evolutionary computation (EC) algorithm. The optimization of each subproblem can be performed independently of each other using a homogeneous or heterogeneous optimizer. Communication between the subproblems is required to build a complete solution to the problem using an objective or fitness function *f*. This implies that a candidate solution in search space $SP^{\left(i\right)}$ contains a few elements (comprising an individual *I*) of the *n*-dimensional problem (I∈SP). Therefore, in CC, a common *n*-dimensional context vector *v* is required to build using a collaborative individual (e.g., the current best individual) from each subproblem. A candidate solution to the problem is built by joining representative collaborators from the context vector to evaluate an individual in a subproblem. Potter and De Jong, in their original CC approach, decomposed an *n*-dimensional problem into *n* 1-dimensional subproblems. In general, the *n*-dimensional problem can be decomposed into *m* subproblems with the same dimension, i.e., $n_{m}=n/m$ [16].

Therefore, a CC consists of three main phases: (1) problem decomposition, (2) subproblem evolution, and (3) collaboration and evaluation [17,18,19]. Problem decomposition involves the process of decomposing a large problem into several subproblems based on the problem structure. Depending on the problem structure, the decomposition can be static or dynamic. When the problem is decomposed statically, it can have one or more elements in each decomposed group. However, the group elements remain fixed throughout the generations. On the other hand, when the problem is decomposed dynamically, the decomposed groups can have different group elements other than the initial generation. Furthermore, the group elements may change in each iteration in the case of dynamic decomposition. Examples of different decomposition methods are in [6,18,20,21]. A homogeneous or heterogeneous evolutionary optimizer can perform subproblem optimization. In addition, the optimization can be carried out sequentially or in parallel. Only one subproblem is evolved in each iteration when optimization is performed sequentially. In contrast, multiple subproblems can be optimized simultaneously in parallel. An example of widely used optimizer in this context is in [22]. At the third stage of a CC, a collaboration mechanism is required to build a complete solution to the problem. The complete solution is then evaluated using the objective function. The collaborative performance of a solution can be assigned as the fitness value to that individual being evaluated. Examples of different collaboration and evaluation models are in [17,23].

### 3.2. Cooperative Co-Evolution-Based Feature Selection with Random Feature Grouping

The cooperative co-evolution-based feature selection with random feature grouping (CCFSRFG) [6] is an evolutionary computation based wrapper FS process that can be described as follows:

For example, a dataset *D* consists of *n* features, i.e., $D=\left\{f_{1},f_{2},f_{3},\ldots ,f_{n}\right\}$. *D*. *D* is decomposed randomly into *m* subdatasets with $s\left(s<n\right)$ features in each subdataset:

$$D_{1}=\left\{f_{i_{1}},f_{i_{2}},\ldots ,f_{i_{s}}\right\},D_{2}=\left\{f_{i_{1}},f_{i_{2}},\ldots ,f_{i_{s}}\right\},\ldots ,D_{m}=\left\{f_{i_{1}},f_{i_{2}},\ldots ,f_{i_{s}}\right\}$$

A linear correlation coefficient can be used for measuring the linear dependency between two random features in a network traffic dataset when the correlations are associated with a dataset’s records linearly. However, in practice, the correlation between the features may be nonlinear for many real-world problems. Hence, the nonlinear dependency between the two features cannot be measured by a correlation study. Alternatively, selecting a subset of features from the dataset that maximize the classification accuracy is more suitable irrespective of whether the dependency between two features is linear or nonlinear [24]. Accordingly, the feature selection framework, CCFSRFG, with FRG as a decomposer, selects a suitable subset of features without considering correlation.

Each subdataset is represented using a subpopulation in CCFSRFG. Here, *s* is the number of features in each individual (i.e., *s* features of a subdataset). Consider the size of each subpopulation (sp) is sz. An example of subpopulation $sp_{1}$ consisting *s* individual can be the following:

$$ind_{1}=\left\{0,1,1,0,\ldots ,1\right\},ind_{2}=\left\{1,1,1,0,\ldots ,0\right\},\ldots ,ind_{sz}=\left\{0,1,1,1,\ldots ,1\right\}$$

A 1 in an individual indicates that the feature in the corresponding is selected for the feature subset selection. However, a 0 indicates that the feature is not selected for the feature subset selection. An individual in any subpopulation is evaluated by combining collaborators (i.e., individuals) from other subpopulations. For example, to evaluate individual $ind_{1}$ in subpopulation $sp_{1}$, a collaborator $ind_{3}$ from subpopulation $sp_{2}$ and a collaborator $ind_{2}$ from subpopulation $sp_{3}$. These three individuals are combined to form a complete solution for the dataset with a reduced number features. Consider a random decomposition of 9 features into three subpopulations $\left(s=4\right)$, is assumed with $sp_{1}\left\{ind_{1}\right\}$ = $\left\{f_{3},f_{9},f_{7},f_{2}\right\}$, $sp_{2}\left\{ind_{2}\right\}$ = $\left\{f_{6},f_{1},f_{5},f_{8}\right\}$, and $sp_{3}\left\{ind_{4}\right\}$ = $\left\{f_{4}\right\}$. If features $\left\{f_{7},f_{2}\right\}$ from $sp_{1}\left\{ind_{1}\right\}$, $\left\{f_{1},f_{5}\right\}$ from $sp_{2}\left\{ind_{2}\right\}$, and $\left\{f_{4}\right\}$ from $sp_{3}\left\{ind_{4}\right\}$ are selected because of a binary (0 or 1) representation of features, the complete solution with sorted feature indices can be defined as follows:

$$solution=\left\{f_{1},f_{2},f_{4},f_{5},f_{7}\right\}$$

The solution with this reduced number of features is then evaluated by the ML classifiers to measure accuracy performance. The best individual with a reduced number of features and the highest classification accuracy is achieved by a penalty-based wrapper objective function introduced in the CCEAFS approach [4].

When there is no previous information available, random collaborators (i.e., individuals) from other subpopulations are used to build a complete solution in the first generation of CCFSRFG. The best individuals from other subpopulations are used as collaborators from generation 1 onwards. The process continues until it reaches a fixed number of generations, until no better fitness is achieved over the generations, or a fixed number of features selected.

## 4. Proposed Methodology

**Figure 4:**
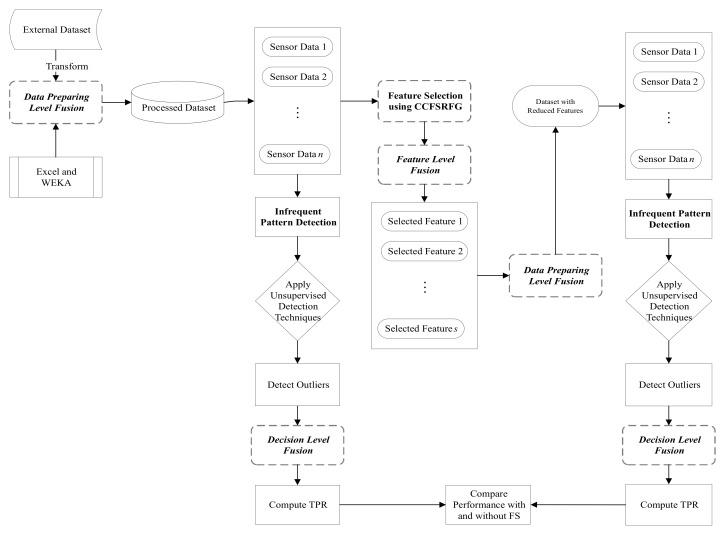
Proposed CCFSRFG-based unsupervised infrequent pattern detection (UIPD) approach with data fusion used in different stages.

The proposed unsupervised infrequent pattern detection (UIPD) is illustrated in Figure 4.

The methodology for UIPD consists of a data preprocessing step utilizing the data fusion methodology. According to the attack category, the data fusion step sorts the data samples, separates the normal and anomalous samples, and reduces the datasets based on selected features after the feature selection (FS) process is applied. Data fusion is also used to prepare the dataset after the outlier is detected via RapidMiner and used for infrequent pattern detection. Microsoft Excel and *WEKA* (https://www.cs.waikato.ac.nz/ml/weka/ (accessed on 16 April 2021)) have been used for this purpose. After the preprocessing of removing attack information from the datasets, the entire dataset was used to compute the outlier using all 10 unsupervised anomaly detection techniques mentioned in Section 2. The infrequent pattern detection performance was computed in terms of TPR and ET. The FS framework, CCFSRFG, was then applied to the dataset to represent it a reduced number of features that maximize classification accuracy. Details of the CCFSRFG process can be found in [6]. In this way, the dataset with the reduced number of features is preprocessed to remove the attack information and the outlier detection is performed using the same 10 unsupervised anomaly detection techniques. Likewise, with the original dataset, the infrequent pattern detection performance was computed in terms of TPR and ET. Finally, the infrequent pattern detection performance was compared with and without FS in terms of TPR. Algorithm 1 is the pseudocode of the proposed UIPD approach using unsupervised anomaly detection techniques. A JAVA-based implementation of UIPD is available at GitHub (https://github.com/bazlurrashid/cooperative-coevolution/tree/UIPD/) (accessed on 16 April 2021).
**Algorithm 1** UIPD**Input:** *.CSV formatted dataset files;**Output:** TPR and ET;1: Using RapidMiner to compute the anomaly scores;2: Sort the anomaly scores in descending order;3: Separate the top instances based on the actual anomalies in the ground truth and store in a CSV file;4: **while** Read the CSV files of actual anomalies and anomalies obtained in previous step until end **do**5:  Split the read lines into columns and store the first column’s values into gInstances[x] and outInstances[x];6:  Increase the value of *x* by 1;7: **end while**8: Compute the number of anomaly instances from both CSV files and store into gSize and outSize, respectively;9: Store the value of outSize−1 into nums array;10: **for**
x=1 to gSize
**do**11:  **for**
y=1 to length of nums
**do**12:   **if**
gInstances[x]==outInstances[nums[y]]
**then**13:    Increase the value of correct by 1;14:    Remove index *y* from the nums array;15:    Jump the execution to the inner loop to continue checking with other index values;16:   **end if**17:  **end for**18: **end for**19: Assign the size of outInstances into anomalies;20: Compute TPR=correct/anomalies;21: Display TPR and ET.


## 5. Experimental Results and Analysis

Experimental results are included in this section and analyzed with and without feature selection (FS) approaches.

### 5.1. Benchmark Dataset

The benchmark UNSW_NB15 (https://www.unsw.adfa.edu.au/unsw-canberra-cyber/cybersecurity/ADFA-NB15-Datasets/ (accessed on 16 April 2021)) dataset used in the experiments is listed in Table 1 with normal and infrequent pattern data distribution. Table 2 lists the infrequent patterns with data samples in the dataset with respect to the total samples and with respect to the anomalous samples, respectively.

The UNSW_NB15 dataset contains a hybrid of the real modern normal and the contemporary synthesized attacks of the network traffic. The dataset is comprised of 9 different attacks, including reconnaissance, backdoor, DoS, exploits, analysis, fuzzers, worms, shellcode, and generic. The dataset has been created to deal with the current network threat environment because the existing benchmark datasets, such as KDD98, KDD99, and NSL-KDD do not (inclusively) include network traffic and modern low footprint attacks. Furthermore, the most used dataset for network traffic analysis is UNWS_NB15 in the last 5 years and KDD99 is more than 20 years old [25].

### 5.2. Parameters and Evaluation Measures

A dynamic decomposition method, called random feature grouping (RFG), the genetic algorithm (GA) as subproblem optimizer, and random and best collaboration model with 1+N have been used for the FS framework CCFSRFG. Subpopulation size: 30, number of subpopulations: 2, and number of features in each subpopulation are 22 and 20, respectively. GA parameters: binary representation, 100% crossover rate, 5% mutation rate, one elitism, and tournament selection. In the case of CCFSRFG termination, 100 successive generations with no improvement have been used. Classification accuracy and true positive rate (TRP) have been used as evaluation measures. The parameters used for different unsupervised anomaly detection techniques using *RapidMiner* (https://rapidminer.com (accessed on 16 April 2021)) are described here. The maximum value of *k*, when required for different anomaly detection techniques, has been selected based on the ceiling of the square root of the total number of instances in a dataset, while the minimum value was kept at 2 [26]. For example, if a dataset has 100 instances, the maximum value of *k* is 10. Mixed measures and mixed Euclidean Distance parameters were set for *k*-NN, LOF, COF, aLOCI, LoOp, INFLO, CBLOF, LDCOF, and CMGOS. For aLOCI, differenceoflevelsL=4,treedepth(levels)=10,numberofgrids=20,nmin=20; for CBLOF, alpha=90.0,beta=5.0; for LDCOF, gamma=0.1; for CMGOS, probabilityfornormalclass=0.975,gamma=0.1,covarianceestimation=Reduction,timestoremoveoutlier=1; for HBOS, parametermode=all. The experimental environment was a desktop computing machine with Intel (R) Core (TM) i7-7700 CPU @3.60 GHz processor, 16.0 GB RAM, and a 64-bit Operating System.

### 5.3. Results and Discussions

A summary of the FS process’s performance results after applying CCFSRFG to the UNSW_NB15 dataset is listed in Table 3 in terms of classification accuracy, the number of features, and execution time (ET). The naïve Bayes classifier and cross-validation were used to evaluate the FS process. The selected 3 features by CCFSRFG from the UNSW_NB15 dataset are proto, service, ct_state_ttl. As a result that CCFSRFG is based on a metaheuristic algorithm, the selected subset of features may not be the same in each execution. It can be expected that there should be a minimum of features in the dataset that can maximize the classification accuracy. However, the selected subset of features by the FS process, such as CCFSRFG, will always depend on how the evolutionary process (selection, crossover, and mutation) is performed internally by the algorithm itself.

From Table 3, it can be observed that CCFSRFG was able to select a suitable subset of features with a very low number of features (only 3 for UNSW_NB15 dataset) compared to the original dataset. Simultaneously, the original accuracy was 45.91 and 92.95, and the accuracy after the FS process was 72.78 and 98.71, respectively. The FS process is computationally expensive, and it also depends on the underlying dataset characteristics. As a result of the 10 different types of attacks in the UNSW_NB15 dataset, the FS process took 9.72 h for the UNSW_NB15 dataset using the available computing resources.

The original UNSW_NB15 dataset and the dataset with a reduced number of features (3 features only) are used for infrequent pattern detection using 10 unsupervised anomaly detection techniques discussed in Section 2. The summary of the experimental results in terms of true positive rate (TPR) is listed in Table 4.

**Figure 5:**
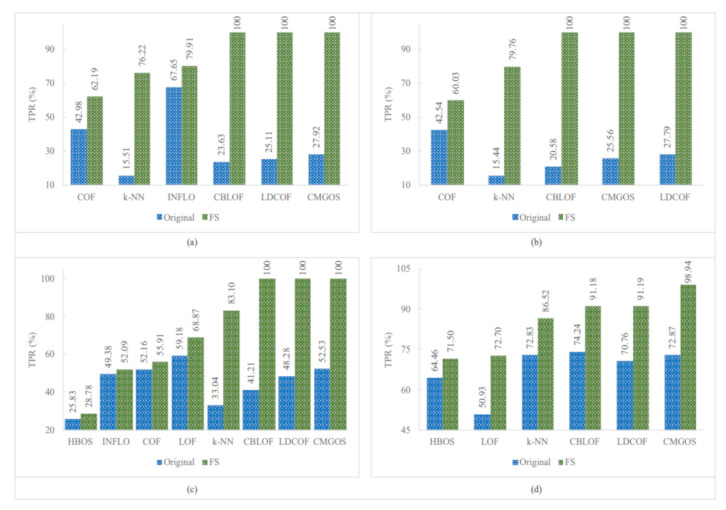
Performance of the individual (unsupervised) infrequent pattern detection techniques with and without FS on UNSW_NB15 dataset for all infrequent patterns: (**a**) Analysis. (**b**) Backdoor. (**c**) DoS. (**d**) Exploits.

**Figure 6:**
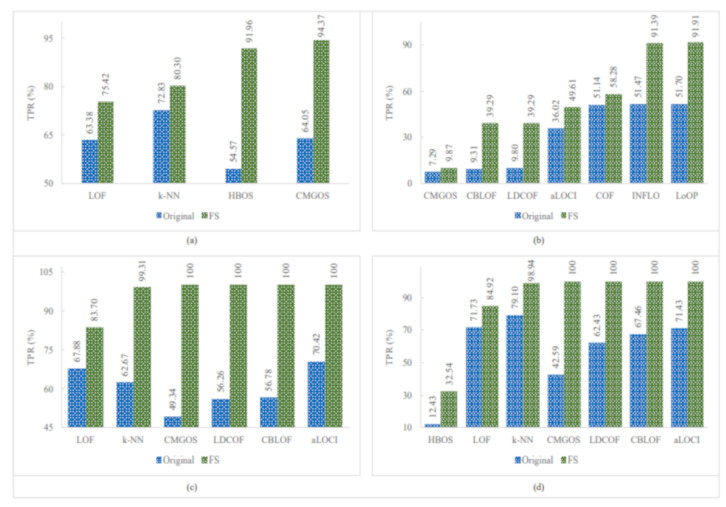
Performance of the individual (unsupervised) infrequent pattern detection techniques with and without FS on UNSW_NB15 dataset for all infrequent patterns: (**a**) Fuzzers. (**b**) Generic. (**c**) Reconnaissance. (**d**) Shellcode.

**Figure 7:**
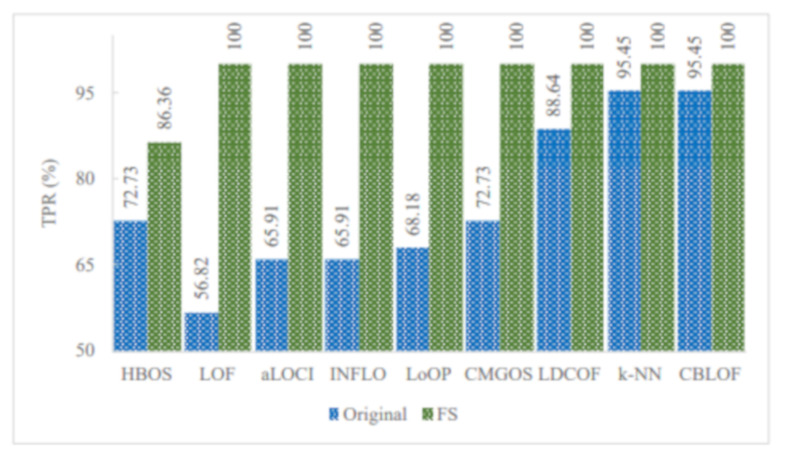
Performance of the individual (unsupervised) infrequent pattern detection techniques with and without FS on UNSW_NB15 dataset for the infrequent pattern: Worms.

Figure 5, Figure 6 and Figure 7 show the improved TPRs from Table 4 by different anomaly detection techniques for detecting the infrequent patterns in UNSW_NB15 dataset. It can be observed that every anomaly detection algorithm improved the TPR for detecting at least two infrequent patterns when using FS. LoOP algorithm was the least, in this case, detecting only two patterns: generic and worms, whereas the CMGOS was the topper in improving TPR for detecting all the patterns. Other algorithms (*k*-NN, LOF, COF, aLOCI, INFLO, CBLOF, LDCOF, and HBOS improved TPRs for 8, 6, 4, 4, 4, 4, 8, 8, and 5 infrequent patterns, respectively. It can also be seen that there were eight anomaly algorithms except for COF and HBOS, which achieved a 100% TPR when using FS for different infrequent pattern detection. First, CBLOF, LDCOF, and CMGOS achieved 100% TPR for detecting analysis, backdoor, DoS, reconnaissance, shellcode, and worms patterns. Second, aLOCI achieved 100% TPR for reconnaissance, shellcode, and worms patterns. Third, *k*-NN, LOF, LoOP, and INFLO achieved a 100% TPR for worms infrequent pattern detection.

**Figure 8:**
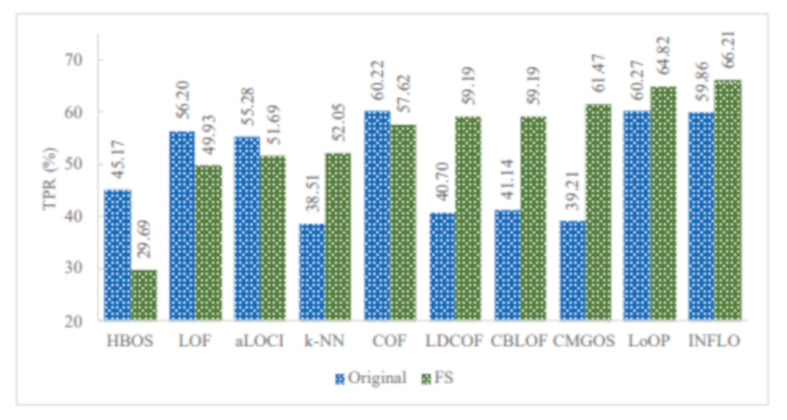
TPR of all infrequent pattern detections with and without FS.

Figure 8 illustrates the TPR of all infrequent pattern detections with and without FS. It can be observed that among the 10 unsupervised anomaly detection algorithms, five algorithms improved the overall TPR in detecting all infrequent patterns when using FS. These algorithms are LDCOF, CBLOF, CMGOS, LoOP, and INFLO. The highest overall TPR achieved was 66.21 by INFLO, while the lowest overall TPR achieved was 29.69 by HBOS when using FS. In the case of the original dataset with all features, the highest overall TPR was 60.27, and the lowest overall TPR was 38.51.

### 5.4. Meaningful Insights

**Figure 9:**
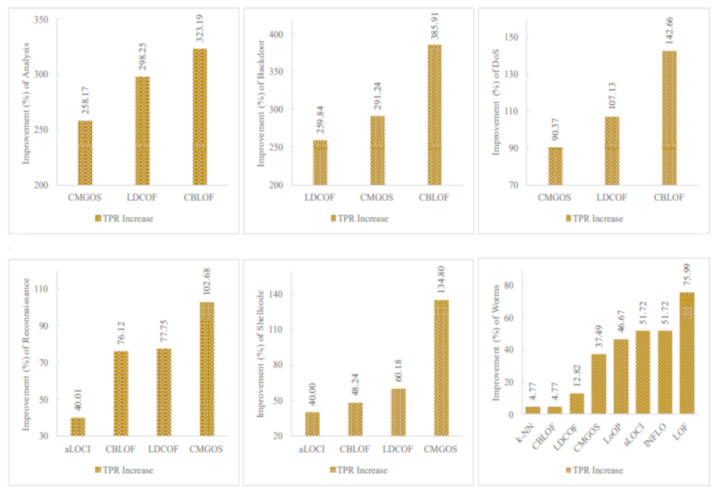
Performance (TPR) improvement for six infrequent patterns by at least three unsupervised anomaly detection techniques where a 100% TPR was achieved when using FS.

The proposed unsupervised infrequent pattern detection (UIPD) has significantly improved infrequent pattern detection performance for at least six patterns: analysis, backdoor, DoS, reconnaissance, shellcode, and worms with a 100% TPR when using the dataset with FS with a reduced number of features. The simulation results of the improvement ratio in TPR with and without FS are displayed in Figure 9. It can be observed that the detection of analysis patterns was significantly improved for three anomaly detection algorithms: CMGOS, LDCOF, and CBLOF. The improved TPR ratios over the original dataset are 258.17%, 298.25%, and 323.19%, respectively. It can be noted that CBLOF, LDCOF, and CMGOS anomaly detection algorithms were the three common algorithms for which the TPR improvement ratio was very significant for all of the above-mentioned six infrequent patterns. The highest TRP ratio improvement over the TPR by the original dataset was 385.91% by CBLOF for the backdoor pattern. On the other hand, the lowest TPR improvement ratio was 4.77% by *k*-NN and CBLOF for worms pattern detection.

**Figure 10:**
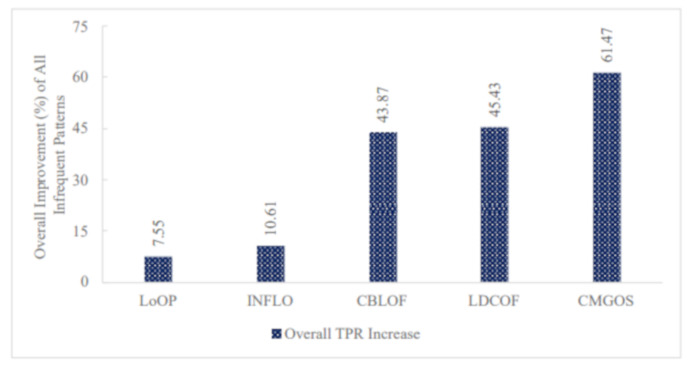
Performance (TPR) improvement of all infrequent patterns by the five unsupervised anomaly detection techniques when using FS.

Furthermore, Figure 10 presents the overall TPR ratio improvement for all infrequent pattern detection by the five anomaly detection algorithms: LoOP, INFLO, CBLOF, LDCOF, and CMGOS when using FS. It can be observed that the highest overall TPR ratio improvement was 61.47% by CMGOS, while the lowest was 7.55% by LoOP.

## 6. Conclusions and Future Work

This paper introduced infrequent pattern detection for reliable network traffic analysis using a robust evolutionary computation approach. For this purpose, a cooperative co-evolution-based feature selection with random feature grouping (CCFSRFG) [6] was used as the feature selection (FS) or feature engineering (FE) mechanism to preprocess the benchmark UNSW_NB15 (https://www.unsw.adfa.edu.au/unsw-canberra-cyber/cybersecurity/ADFA-NB15-Datasets/ (accessed on 16 April 2021)) dataset. The original dataset and the dataset with a reduced number of features after applying the CCFSRFG were used for infrequent pattern detection. Ten unsupervised anomaly detection techniques were used to evaluate infrequent pattern detection performance in terms of TPR. Comparisons of the performance results were shown with and without FS. Although the FS is computationally expensive, it was shown that if a suitable FS process is applied before the detection of infrequent pattern, there can certainly be a few anomaly detection techniques that can improve the TPR for a few infrequent patterns. The actual execution time for infrequent pattern detection with an FS process will always depend on datasets’ complexities, including the number of features, number of instances, and the data themselves. Therefore, as future work, the proposed UIPD approach of infrequent pattern detection for reliable network traffic analysis can be investigated on other datasets and also using different base classifiers other than naïve Bayes for the FS process.

## Figures and Tables

**Table 1:** Distribution of normal and anomalous data.

Dataset	Normal (%)	Anomalous (%)	No. of Instances	No. of Features
UNSW_NB15	44.94	55.06	82,332	42

**Table 2:** Distribution of infrequent pattern data in UNSW_NB15 dataset.

Anomaly	Weight (%)	Anomalous (%)	No. of Instances
Analysis	00.82	01.49	677
Backdoor	00.71	01.29	583
DoS	04.97	09.02	4,089
Exploits	13.52	24.56	11,132
Fuzzers	07.36	13.37	6,062
Generic	22.92	41.63	18,871
Reconnaissance	04.25	07.71	3,496
Shellcode	00.46	00.83	378
Worms	00.05	00.10	44

*Note: “Weight” indicates the (%) of data samples with respect to the total samples in the dataset. “Anomalous” indicates the (%) of data samples with respect to the anomaly samples in the dataset.*

**Table 3:** Summary of results for UNSW_NB15 dataset with and without FS using a naïve Bayes classifier.

	Without FS		With FS		
Dataset	Accuracy (%)	No. of Features	Accuracy (%)	No. of Features	Execution Time (hour)
UNSW_NB15	45.91	42	72.78	3	09.72

**Table 4:** Summary of performance of the individual unsupervised infrequent pattern detection in terms of TPR (%) with and without FS for UNSW_NB15 dataset.

Infrequent Pattern	With or Without FS	Outlier									
		*k*-NN	LOF	COF	aLOCI	LoOP	INFLO	CBLOF	LDCOF	CMGOS	HBOS
Analysis	Ori	15.51	68.83	42.98	72.38	93.94	67.65	23.63	25.11	27.92	13.88
	FS	**76.22**	40.77	**62.19**	26.44	56.28	**79.91**	**100**	**100**	**100**	08.57
Backdoor	Ori	15.44	54.72	42.54	66.55	74.27	66.38	20.58	27.79	25.56	11.84
	FS	**79.76**	50.77	**60.03**	40.99	61.06	63.12	**100**	**100**	**100**	10.46
DoS	Ori	33.04	59.18	52.16	68.11	52.31	49.38	41.21	48.28	52.53	25.83
	FS	**83.10**	**68.87**	**55.91**	58.38	49.79	**52.09**	**100**	**100**	**100**	**28.78**
Exploits	Ori	72.83	50.93	67.58	68.03	65.11	62.95	74.24	70.76	72.87	64.46
	FS	**86.52**	**72.70**	57.72	41.16	46.53	44.98	**91.18**	**91.19**	**98.94**	**71.50**
Fuzzers	Ori	72.83	63.38	74.02	70.37	74.07	74.83	72.29	68.87	64.05	54.57
	FS	**80.30**	**75.42**	57.34	45.61	43.29	48.19	00.00	00.00	**94.37**	**91.96**
Generic	Ori	04.54	53.48	51.14	36.02	51.70	51.47	09.31	09.80	07.29	42.48
	FS	04.39	17.44	**58.28**	**49.61**	**91.91**	**91.39**	**39.29**	**39.29**	**09.87**	00.61
Reconnaissance	Ori	62.67	67.88	74.57	70.42	72.03	76.74	56.78	56.26	49.34	19.45
	FS	**99.31**	**83.70**	56.64	**100**	36.87	47.08	**100**	**100**	**100**	04.92
Shellcode	Ori	79.10	71.43	84.92	71.43	80.95	80.69	67.46	62.43	42.59	12.43
	FS	**98.94**	**84.92**	49.21	**100**	34.66	29.89	**100**	**100**	**100**	**32.54**
Worms	Ori	95.45	56.82	81.82	65.91	68.18	65.91	95.45	88.64	72.73	72.73
	FS	**100**	**100**	00.00	**100**	**100**	**100**	**100**	**100**	**100**	**86.36**

*Note: “Ori” indicates TPR (%) without FS, “FS” indicates TPR (%) with FS. The bold values represent the improvements in detecting infrequent patterns with FS by the corresponding techniques.*

## Data Availability

Not applicable.
